# Circulating miR-765 and miR-149: Potential Noninvasive Diagnostic Biomarkers for Geriatric Coronary Artery Disease Patients

**DOI:** 10.1155/2015/740301

**Published:** 2015-01-15

**Authors:** Md Sayed Ali Sheikh, Ke Xia, Fei Li, Xu Deng, Umme Salma, Hai Deng, Liu Wei Wei, Tian-Lun Yang, Jun Peng

**Affiliations:** ^1^Department of Cardiology, Xiangya Hospital, Central South University, No. 87 Xiangya Road, Changsha, Hunan 410078, China; ^2^Center for Vascular Biology and Inflammation, Cardiovascular Division, Department of Medicine, Brigham and Women's Hospital, Harvard Medical School, Boston, USA; ^3^Department of Gynecology and Obstetrics, Xiangya 3rd Hospital, Central South University, Changsha, China; ^4^Department of Pharmacology, School of Pharmaceutical Sciences, Central South University, Changsha 410078, China

## Abstract

The purpose of this study was to evaluate the diagnostic value of circulating miR-765 and miR-149 as noninvasive early biomarkers for geriatric coronary artery disease (CAD) patients. A total of 69 angiographically documented CAD patients including 37 stable CAD (72.9 ± 4.2 years) and 32 unstable CAD (72.03 ± 4.3 years) and 20 healthy subjects (71.7 ± 5.2 years), matched for age, sex, smoking habit, hypertension, and diabetes, were enrolled in this study. Compared with healthy subjects, circulating miR-765 levels were increased by 2.9-fold in stable CAD and 5.8-fold in unstable CAD patients, respectively, while circulating miR-149 levels were downregulated by 3.5-fold in stable CAD and 4.2-fold in unstable CAD patients, respectively. Furthermore, plasma levels of miR-765 were found to be positively correlated with ages within control, stable, and unstable groups. The ROC curves of miR-765 and miR-149 represented significant diagnostic values with an area under curve (AUC) of 0.959, 0.972 and 0.938, 0.977 in stable CAD patients and unstable CAD patients as compared with healthy subjects, respectively. Plasma levels of miR-765 and miR-149 might be used as noninvasive biomarkers for the diagnosis of CAD in geriatric people.

## 1. Introduction

Coronary artery disease (CAD) is the leading cause of morbidity and mortality in the world. On an average, CAD caused 1 of every 6 deaths in the United States. Approximately, 80% of people who die of CAD are ≥65 years of age. Prevalence of CAD will increase ≈18% by 2030 [1]. Early diagnosis of CAD has an important role in patient management. In recent 30 years, big progress has been made to improve the diagnosis, treatment, and prognosis for CAD and to reduce the morbidity and mortality rate. Coronary angiogram (CAG) is the well-established invasive method for diagnosis of CAD. However, there is still a clinical need for novel diagnostic noninvasive biomarker and new therapeutic interventions to decrease CAD incidence. Circulating miRNAs seem to be promising highly sensitive novel noninvasive biomarkers for early diagnosis of CAD [2, 3].

MiRNAs are highly specific, endogenous, small (~22 nucleotides), single-stranded, noncoding RNAs that regulate gene expression at the posttranscriptional level by binding to the 3′ untranslated region (UTR) through their target mRNAs [4]. MiRNAs are critically involved during cardiogenesis as well as progression of CAD [5]. In fact, miRNAs are now well recognized as key regulatory molecules in endothelial cells (ECs), vascular smooth muscle cells (VSMCs), platelets, and immune cells that contribute to the initiation and progression of atherosclerosis [6]. It has been revealed that several miRNAs (miR-10a, miR-19a, miR-23b, miR-17-92, miR-21, miR-24, miR-92a, miR-101, miR-126, miR-145, miR-155, miR-205, miR-663, and miR-712) are significantly expressed in the vasculature and show altered expression during various vascular disorders, such as vascular injury, atherosclerosis, angiogenesis, and arterial remodeling [7, 8], whereas miR-29b, miR-24, and miR-365 play a key role in prevention of atherosclerosis through modifying their targets [9–11]. MiRNAs that are present in serum or plasma are collectively called circulating miRNAs, which are extremely stable in boiling water, prolonged room temperature incubation, or repetitive freezing-thawing cycles and highly resistant to plasma RNase activity due to internalization in vesicles and binding to circulating proteins [12, 13]. Several recent studies have reported that circulating miRNAs expression levels altered in patients with stable coronary artery disease (SA) [14], unstable angina (UA) [15], acute coronary syndrome (ACS) [16], acute myocardial infarction (AMI) [17], and heart failure [18].

In addition, some circulating miRNAs (such as miR-208b, miR-499, miR-1, miR-126, miR-423-5p, miR-142-3p, miR-486-3p, miR-150-3p, miR-26a-5p, and miR-191-5p) have been identified as novel biomarkers for diagnosis of AMI and HF [19–23]. However, there is limited information on the value of circulating miRNAs as noninvasive biomarkers for diagnosis of CAD in geriatric patient.

Therefore, in the present study, we assessed the plasma levels of miR-765 and miR-149 in geriatric patients with CAD to see whether they could be used as novel noninvasive biomarkers for diagnosis of CAD.

## 2. Materials and Methods

### 2.1. CAD Subjects

The protocol of this study was supported according to the principles of the Declaration of Helsinki. The study was approved by the Medical Ethics Committee of Xiangya Hospital, Central South University, Hunan, China. Written informed consent was obtained from all the participants at the time of enrollment.

Angiographically, documented sixty-nine CAD patients were enrolled in this study from cardiology department of Xiang Ya Hospital between July 2012 and September 2013. CAD was defined as at least one major epicardial vessel with ≥50% stenosis, assessed by quantitative coronary angiography, and evaluated independently by two operators, who made visual estimation of luminal narrowing in multiple segments based on a modified form of the AHA/ACC classification of the coronary tree.

CAD was also categorized as either stable or unstable according to ACC/AHA guidelines [24, 25]. Stable coronary artery disease patients showed typical chest pain on exertion associated with ST segment depression >1.0 mm on an exercise tolerance test (ETT). However, unstable coronary artery disease was defined as chest pain occurring at rest or minimal exertion and usually lasting >20 minutes, recent onset (within one month) or with a crescendo pattern (i.e., more severe, prolonged, or frequent than previously), without elevated myocardial necrosis related cardiac serum markers including creatine kinase (CK-MB) and troponin I or T levels. The inclusion criteria of CAD subjects were age of 65 to 85 years and CAD confirmed by CAG. The exclusion criteria of CAD subjects were as follows: acute myocardial infarction (AMI); elevated cardiac troponin I (cTnI) or creatine kinase (CK-MB) levels; impaired left ventricular ejection fraction (LVEF) ≤45%; congestive heart failure; severe hepatic and renal dysfunction; and ongoing inflammatory and malignant disease.

### 2.2. Healthy Subjects

Twenty healthy subjects matched for age, sex, smoking habit, hypertension, and diabetes were recruited in this study. The criteria for controls were as follows: age of 65 to 85 years, ECG, ETT, and echocardiogram reports within normal limit, no history of CAD or stroke, without evidence of acute or chronic hepatic and renal disease, and not be hospitalized for at least 4 months prior to participation. All subjects provided written informed consent at the time of enrollment.

### 2.3. Plasma Samples Collection

Peripheral 5 mL venous blood samples were collected in EDTA coated tubes from patients and healthy subjects at Xiang Ya Hospital and processed within 30 min. Plasma was prepared following a two-step centrifugation procedure. After plasma separation from blood, samples were first centrifuged at 1.500 ×g for 15′ at 4°C. The supernatant was collected and then centrifuged again at 14.000 ×g for 15′ at 4°C to obtain pure plasma and subsequently supernatant was transferred to RNase-free tubes and stored at −80°C until use.

### 2.4. RNA Extraction from Plasma

Total RNA was isolated by using a TRIzol-based miRNA isolation protocol (Invitrogen). Firstly, 250 *μ*L of plasma was mixed briefly with 750 *μ*L of TRIzol, incubated for 5 min at room temperature (RT), and then mixed with 200 *μ*L chloroform, incubated for 3 min at RT. The aqueous, inter, and organic phase were separated by centrifugation at 4°C, at 12,000 rmp for 15 min. Secondly, the upper aqueous phase was collected and subsequently mixed with 500 *μ*L of 100% isopropanol and incubated at −20°C for overnight and after that centrifuged at 4°C, at 13000 rmp for 15 min for precipitation. RNA samples were washed 2 times with 500 *μ*L of 80% ethanol and centrifuged again at 4°C at 7500 rmp for 10 min. Finally, supernatants were eliminated and dried for 5 min. Then, RNA samples were dissolved in 30 *μ*L of RNAse-free (DEPC) water and incubated for overnight (8 hours) at 4°C. Afterward, the RNA concentrations were quantified with a NanoDrop ND-1000 spectrophotometer (NanoDrop Technologies Inc., Wilmington, USA) and stored at −80°C for future use.

### 2.5. miRNAs Expression Analysis by Quantitative PCR

We used real-time quantitative reverse-transcription PCR (qRT-PCR) to validate the miRANs expression. Initially, 4 *μ*L of total pure RNA was reverse-transcribed (RT) to cDNA at 42°C for 30 minutes using miRNA-specific reverse transcription kits (RiboBio, Guangzhou, China) according to the instructions of the manufacturer, using a RT-PCR System (BIO-RAD, USA). Subsequently, 2 *μ*L of cDNA was used as the template in real-time quantitative PCR reaction. Plasma miR-765 and miR-149 expression were measured using SYBR Green miRNA quantitative reverse transcription polymerase chain reaction kits (Takara, Dalian, China) according to the manufacturer's protocol, using a 7300 Real-Time PCR System (Applied Biosystems, CA, USA). Melting curve analysis was performed at the end of the PCR cycles in order to confirm the specificity of the expected PCR product.

miR-156a was used as the normalization control. PCR was performed in triplicate for each sample for both control and each miRNA at the same time. The Ct (cycle threshold) values were determined using SDS2.1 software. The relative expression of specific miRNA was calculated by the comparative Ct method, which was defined as 2^−ΔCt^, ΔCt = (Ct miRNA of sample *x* − Ct 156aRNA of sample *x*). The Ct values from qRT-PCR assays between 15 and 35 were considered to be expressed. To minimize the number of errors, we only considered those miRNAs whose expression in CAD and unstable CAD patients significantly differed from the controls at least more than 2-fold.

### 2.6. Clinical and Laboratory Assays

Plasma cardiac troponin I (cTnI), creatine kinase (CK-MB), and lactate dehydrogenase (LDH) levels were measured using the Access Immunoassay System (Beckman Coulter). Fasting blood sugar (FBS), triglycerides (TG), total cholesterol (TC), low-density lipoprotein cholesterol (LDL-C), high-density lipoprotein cholesterol (HDL-C), aspartate aminotransferase (AST), alanine aminotransferase (ALT), blood urea nitrogen (BUN), creatinine (Cr), uric acid (UA), and high-sensitivity C-reactive protein (hs-CRP) were measured by automatic analyzer (Hitach75, Tokyo, Japan). Clinical history, physical examination, serial 12-lead ECG, echocardiogram, and medication records were also collected.

### 2.7. Statistical Analysis

Clinical data were analyzed with SPSS software (version 16.0; SPSS, Chicago, IL) and presented as mean ± standard deviation (SD). Circulating miRNAs expression data were analyzed and graphs were constructed by GraphPad Prism version 6 for Windows (GraphPad Software, San Diego, CA, USA) and reported as mean ± standard error of the mean (SEM). For continuous variables among groups, Student's *t*-test, the Mann-Whitney test, One-way ANOVA, or nonparametric Kruskal-Wallis test was used as appropriate. For categorical variables, Fisher's exact test or the chi-square (*χ* ^2^) test was used. The correlations between parameters were measured with Spearman rank correlation or Pearson correlation. The receiver operating characteristic (ROC) curves were used for discriminating CAD patients from the healthy subjects. All *P* values are two-sided and *P* < 0.05 was considered statistically significant.

## 3. Results

### 3.1. Clinical Characteristics of the Study Subjects

A total of 69 consecutive CAD patients, 37 patients with stable CAD (20 males and 17 females, 72.9 ± 4.2 years) and 32 patients with unstable CAD (18 males and 14 females, 72.03 ± 4.3 years), and 20 healthy subjects (10 males and 10 females, 71.7 ± 5.2 years) matched for age, sex, smoking habit, hypertension, and diabetes were enrolled in this study. BMI, FBS, SBP, DBP, TG, TC, HDL, LDL, AST, ALT, Cr, cTnI, CK-MB, LDH, LVEF, history of smoking, diabetes, hypertension, and treatment records were collected, respectively. There were no significant statistical differences between stable CAD group and unstable CAD group (*P* > 0.05). However, hs-CRP levels (16.3 ± 5.3 mg/L) were significantly (*P* < 0.001) higher in unstable CAD group compared with stable CAD group (14.2 ± 3.3 mg/L). The details of clinical characteristics of the study subjects were shown in Table 1.

### 3.2. Expression Pattern of Circulating miR-765 and miR-149 Levels in Coronary Artery Disease Patients

**Figure 1:**
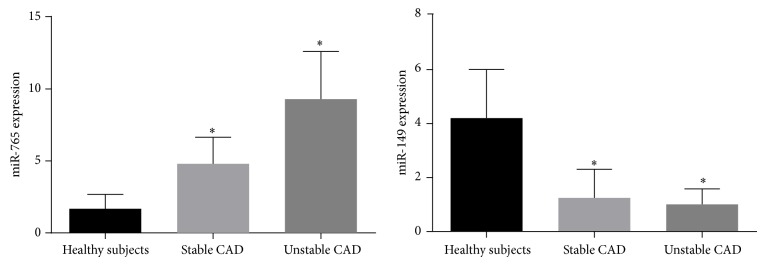
Plasma levels of circulating miR-765 and miR-149 in stable and unstable CAD patients.

We measured miR-149 and miR-765 levels in plasma from stable and unstable coronary artery disease patients to determine whether circulating miRNAs levels were correlated with CAD or not (Figure 1). We found that circulating miR-765 levels were significantly increased by 2.9-fold in stable CAD and 5.8-fold in unstable CAD patients, respectively, compared with healthy subjects (*P* < 0.001) while circulating miR-149 levels were significantly downregulated by 3.5-fold in stable CAD and 4.2-fold in unstable CAD patients, respectively (*P* < 0.001).

### 3.3. Diagnostic Value of Circulating miRNAs in CAD Patients

**Figure 2:**
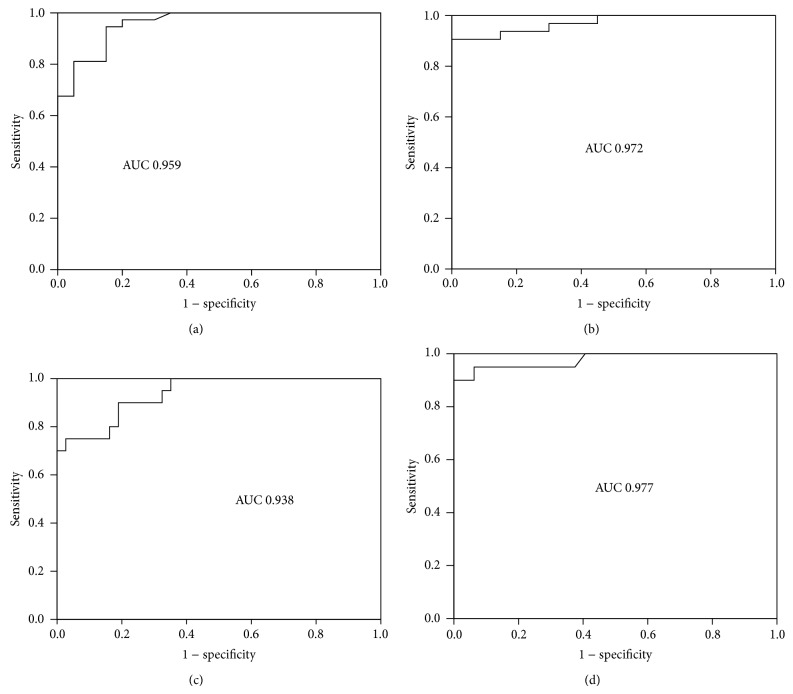
Diagnostic value of circulating miR-765 and miR-149 was analyzed by ROC curve. (a) ROC curve of miR-765 between control group and stable group. The area under curve (AUC) is 0.959. (b) ROC curve of miR-765 between control group and unstable group. AUC is 0.972. (c) ROC curve of miR-149 between control group and stable group. AUC is 0.938. (d) ROC curve of miR-149 between control group and unstable group. AUC is 0.977.

To investigate the diagnostic accuracy of circulatory miRNAs (miR-765, miR-149) as potential biomarkers of CAD, receiver operating characteristics (ROC) curve analysis was performed. The ROC curve of miR-765 reflected a significant separation between stable CAD patients and controls and unstable CAD patients and controls, with an area under curve (AUC) of 0.959 and 0.972, respectively, for the diagnosis of CAD patients (Figures 2(a) and 2(b)). Similarly, The ROC curve of miR-149 also showed a strong ability to distinguish between stable CAD patients and controls and unstable CAD patients and controls, with an area under curve (AUC) of 0.938 and 0.977, respectively, for the identification of CAD patients (Figures 2(c) and 2(d)). It was suggested that circulating miR-765 and miR-149 might be used as the potential biomarkers for early diagnosis of CAD in geriatric patients.

### 3.4. Role of Age Variation of miR-765 and miR-149 in Different Groups

We examined plasma miRNAs expression at different ages (Table 2); we found that plasma levels of miR-765 were significantly (*P* < 0.05) correlated with different ages within control, stable, and unstable groups. In contrast, plasma levels of miR-149 among three groups with different ages were not statistically significant.

## 4. Discussion

CAD is the most common form of cardiovascular disease. An early and correct diagnosis can assure urgent initiation of reperfusion therapy to potentially decrease the morbidity and mortality rate of CAD. Based on their rapid release and stability in plasma, some circulating miRNAs such as miR-208b, miR-499, miR-1, and miR-133 have been demonstrated as novel diagnostic biomarkers in patients with acute myocardial infarction and acute coronary syndrome [2, 19, 26–28]. Circulating miR-30a, miR-126, and let-7b might be useful biomarkers in patients with ischemic stroke [29], whereas circulating miRNAs (miR-16, miR-25) serve as novel biological markers for the diagnosis of intracranial aneurysms (IAs) [30]. Circulating miR-451 and miR-1246 may be considered potential biomarkers for early detection of mild-to-severe human pulmonary hypertension (PH) [31], while circulating levels of miR-21, miR-126, miR-155, and miR-210 may be used as useful biomarkers for the diagnosis of essential hypertension patients [32]. Very recently, it has been found that serum levels of miR-10a, miR-31, miR-92a, and miR-155 have strong correlation with tissue expression and can be used as noninvasive biomarkers for heart transplant rejection [33]. Plasma levels of miR-483-3p and miR-21 are highly expressed in pancreatic cancer patients compared with controls [34]. Circulating miR-133a, miR-208a, miR-31, and miR-135a levels were significantly upregulated and miR-126, miR-17, miR-92a, and miR-155 levels were obviously downregulated in coronary artery disease patients [8, 35].

However, circulating miRNAs levels in geriatric CAD patients have not been previously evaluated. We have chosen geriatric patients into our work due to the following reasons: (i) CAD is very common after the age of 65 years; (ii) elderly CAD patients frequently present with atypical symptoms such as dyspnea and/or epigastric discomfort without chest pain, and uninterpretable ECG, as a result, is a challenging problem to diagnose CAD; (iii) though invasive CAG techniques are the best method for diagnosis of CAD, it has various complications. The present study investigated the plasma levels of circulating miR-765 and miR-149 to establish their potential role as CAD biomarkers in elderly patients. In the present work, we found that plasma level of miR-765 was significantly (*P* < 0.001) elevated, while plasma level of miR-149 was remarkably (*P* < 0.001) decreased in stable and unstable coronary artery disease patients compared with healthy subjects, respectively.

It has been reported that circulating miR-765 levels were markedly increased in patients with traumatic brain injury (TBI) and pregnant women with neural tube defects (NTDs) fetuses, suggesting that miR-765 may be used as a useful clinical biomarker for diagnosis of TBI and NTDs [36, 37]. van Rooij et al. found that miR-149 was significantly downregulated in AMI, whereas Wu et al. established that human-miR-149 was strongly associated with increased risk of CAD in Chinese Han population [38, 39].

Furthermore, we explored the diagnostic potential of miRNA-765 and miR-149 by calculating AUC values from the ROC curve. We found that AUC values of miR-765 and miR-149 were significantly higher 0.959, 0.972, 0.938, and 0.977 in stable CAD patients and unstable CAD patients as compared with healthy subjects, respectively. These findings suggested that miR-765 and miR-149 can be used as potential noninvasive biomarkers for diagnosis of CAD patients. Our study also found that plasma levels of miR-765 were significantly (*P* < 0.005) correlated with age but plasma levels of miR-149 among three groups with different ages were not statistically significant. Recently, it has been demonstrated that miRNAs have significant association with ageing heart [40, 41].

To minimize possible bias from patient selection, subjects with similar age, gender, total cholesterol, total glyceride, high-density lipoprotein, low-density lipoprotein, systolic blood pressure, diastolic blood pressure, aspartate aminotransferase, alanine aminotransferase, creatinine, cardiac troponin I, creatine Kinase-MB, lactate dehydrogenase, left ventricular ejection fraction, diabetes mellitus, smoking, hypertension, and medication history were recorded in our study. Statistical results showed that they have no influence on plasma miR-765 and miR-149 levels. These data indicated that miR-765 and miR-149 may be potential biomarkers for the diagnosis of CAD.

To reduce possible errors derived from qRT-PCR assays, in preresearch (*n* = 15), we were using three potential endogenous control miRNAs (U6, miR-cel-39, and miR-156a); among them we had chosen miR-156a as a standard inner control. Depending on our own experience, the usage of synthetic mimic miR-156 instead of commonly used cel-miR-39 during RNA extraction from plasma of CAD patients can achieve much higher quality of total RNA (OD ratio: 1.8–2.2, nucleic acid concentration 50–500 *μ*g). In addition, the usage of endogenous miR-156 instead of commonly used U6 for endogenous control during real time-PCR can obtain more reliable results (Ct value: 18–25) [2, 42]. Furthermore, Ct values of miRNAs expression >35 were not included in our study. Therefore, our results revealed that expression of plasma miR-765 and miR-149 levels was more stable and reliable in the healthy subjects and study groups.

In our study, we demonstrated that hs-CRP levels were significantly increased in stable CAD and unstable CAD patients compared with healthy subjects. High-sensitivity C-reactive protein is used mainly as a marker of inflammation and it is slightly increasing with aging. C-reactive protein levels are positively associated with CAD [43]. Generally, CAD is caused by atherosclerosis. Atherosclerosis is a hyperlipidemia-induced chronic inflammatory process of the arterial wall, involving interleukins (ILs) such as IL-1*β*, IL-6, tumor necrosis factor (TNF)-*α*, and C-reactive protein (CRP) [44]. Several miRNAs are directly or indirectly regulating endothelial dysfunction and atherosclerosis through their targets such as miR-1 which prevents high-cholesterol induced endothelial dysfunction through myosin light chain kinase (MLCK) expression and extracellular signal regulated kinase (ERK) phosphorylation [45]. MiR-29b suppressed the proliferation and migration of SMCs and prevents atherosclerosis through the inhibition of their targets Mcl-1 and MMP2 [9]. It has been recommended that C-reactive protein levels are significantly decreased in CAD patients following high-intensity statin therapy [46]. However, the relationship between miRNAs and high-sensitivity C-reactive protein is still unknown and needs further study.

The present study provides the first clinical evidence of circulating miR-765 and miR-149 as noninvasive biomarkers for CAD patients. However, several limitations of the present work need to be mentioned. Firstly and most importantly, the present work represents a single-center study using a small sample size of CAD patients. Multicenter large-scale clinical studies will be needed to confirm the potential value of circulating miRNAs as noninvasive diagnostic biomarkers for CAD patients. Secondly, our study was unable to detect the mechanism of upregulation and downregulation of miR-765 and miR-149 in geriatric CAD patients. Finally, measurement of plasma miRNAs requires qRT-PCR, which is expensive and time-consuming. Therefore, less expensive and newer techniques to detect plasma miRNA levels more rapidly can be expected in the near future.

## 5. Conclusion

Our results suggested that plasma levels of miR-765 and miR-149 could be potential noninvasive biomarkers for the diagnosis of geriatric CAD patients.

## Figures and Tables

**Table 1:** Clinical characteristics of the study subjects.

Characteristics	Controls (*n* = 20)	Stable CAD (*n* = 37)	Unstable CAD (*n* = 32)	*P*_1_	*P*_2_	*P*_3_
Age (years)	71.7 ± 5.2	72.97 ± 4.28	72.03 ± 4.36	0.335	0.829	0.370
Male/female	10/10	25/12	18/14	0.257	0.777	0.333
BMI (kg/m^2^)	22.29 ± 1.49	23.08 ± 3.03	24.38 ± 3.46	0.334	0.015	0.072
Smoker (%)	55	64.8	75	0.571	0.224	0.362
Hypertension (%)	50	70.2	78.1	0.158	0.067	0.585
Dyslipidemia (%)	30	51.3	50	0.165	0.249	0.911
DM (%)	15%	24.3%	34.3	0.510	0.200	0.359
FBS	4.79 ± 0.43	5.04 ± 1.13	5.54 ± 1.04	0.642	0.026	0.098
SBP (mmHg)	133.05 ± 7.30	141.4 ± 16.17	137.8 ± 18.12	0.137	0.538	0.606
DBP (mmHg)	77.8 ± 4.13	78.48 ± 7.68	79.71 ± 6.80	0.734	0.332	0.450
TG (mmol/L)	1.42 ± 0.78	1.67 ± 0.73	1.87 ± 1.03	0.305	0.072	0.336
TC (mmol/L)	4.32 ± 1.38	4.4 ± 1.1	4.51 ± 1.18	0.805	0.566	0.694
HDL (mmol/L)	1.21 ± 0.28	1.18 ± 0.37	1.21 ± 0.28	0.729	0.976	0.718
LDL (mmol/L)	2.65 ± 1.29	3.13 ± 1.26	3.25 ± 1.61	0.221	0.139	0.731
hs-CRP (mg/L)	0.7 ± 0.48	14.2 ± 3.3	16.3 ± 5.3	<0.001	<0.001	0.025^*^
AST	15 ± 8.75	18.7 ± 10.1	17.9 ± 9.2	0.154	0.274	0.723
ALT	16.6 ± 8.99	18.4 ± 9.5	20.8 ± 9.9	0.492	0.119	0.290
Cr	82.6 ± 17.72	84 ± 18.8	86.6 ± 18.8	0.793	0.453	0.558
cTnI	0	0.05 ± 0.02	0.06 ± 0.03	<0.001	<0.001	0.095
CK-MB	0	12.1 ± 5.1	13.8 ± 3.5	<0.001	<0.001	0.086
LDH	0	198 ± 33.1	209.4 ± 52.9	<0.001	<0.001	0.220
LVEF, (%)	67.3 ± 5.14	64.1 ± 6.2	62.6 ± 7.2	0.075	0.011	0.331
Aspirin, (%)	30	45.9%	50%	0.273	0.249	0.737
Nitrates, (%)	20	40.5	46.8	0.148	0.076	0.597
*β*-blocker, (%)	35	62.1	65.6	0.421	0.046	0.765
CCB, (%)	25	35.1	31.2	0.389	0.757	0.567
ACEI or ARB, (%)	30	35.1	37.5	0.772	0.766	0.211
Statin, (%)	35	54.1	43.7	0.266	0.575	0.393
Diuretic, (%)	15	27.2	21.8	0.346	0.722	0.620
PCI, (%)	0	21.6	28.1	*≠*	*≠*	0.532

Data reported as mean ± SD. BMI, body mass index; DM, diabetes mellitus; FBS, fasting blood sugar; SBP, systolic blood pressure; DBP, diastolic blood pressure; TC, total cholesterol; TG, total glyceride; HDL, high-density lipoprotein; LDL, low-density lipoprotein; hs-CRP, high sensitivity-C-reactive protein; AST, aspartate aminotransferase; ALT, alanine aminotransferase; Cr, creatinine; cTnI, cardiac troponin I; CK-MB, creatine Kinase-MB; LDH, lactate dehydrogenase; LVEF, left ventricular ejection fraction; CCB, calcium channel blocker; ACEI, angiotensin-converting enzyme inhibitor; ARB, angiotensin receptor blocker; and PCI, percutaneous coronary intervention. All *P* values are represented as comparisons between stable CAD patients and unstable CAD patients. Mann-Whitney test was performed for continuous variables and *χ* ^2^ test was performed for categorical variables. *P*_1_ value (healthy subjects versus stable CAD patients), *P*_2_ value (healthy subjects versus unstable CAD patients), and *P*_3_ value (stable CAD patients versus unstable CAD patients), ^*^ *P* < 0.05.

**Table 2:** Plasma levels of miR-765 and miR-149 in different groups with different ages.

	Control	Stable CAD	Unstable CAD
miR-765			
65–74 years	1.394 ± 0.842	4.246 ± 1.754	8.474 ± 3.208
75–85 years	2.816 ± 0.679	5.842 ± 1.410	11.104 ± 2.822
*P* value	0.014	0.008	0.034
miR-149			
65–74 years	4.210 ± 1.56	1.19 ± 0.933	1.020 ± 0.578
75–85 years	4.489 ± 2.419	1.36 ± 1.049	0.979 ± 0.718
*P* value	0.763	0.594	0.864

Values were reported with mean ± SD.

## References

[1] Go A. S., Mozaffarian D., Roger V. L. (2013). Heart disease and stroke statistics 2013 update: a report from the American Heart Association. *Circulation*.

[2] Sayed A. S. M., Xia K., Salma U., Yang T., Peng J. (2014). Diagnosis, prognosis and therapeutic role of circulating miRNAs in cardiovascular diseases. *Heart, Lung and Circulation*.

[3] Lindahl B. (2013). Acute coronary syndrome—the present and future role of biomarkers. *Clinical Chemistry and Laboratory Medicine*.

[4] Baek D., Villén J., Shin C., Camargo F. D., Gygi S. P., Bartel D. P. (2008). The impact of microRNAs on protein output. *Nature*.

[5] Thum T., Catalucci D., Bauersachs J. (2008). MicroRNAs: novel regulators in cardiac development and disease. *Cardiovascular Research*.

[6] Alexy T., Rooney K., Weber M. (2014). TNF-*α* alters the release and transfer of microparticle -encapsulated miRNAs from endothelial cells. *Physiological Genomics*.

[7] Dangwal S., Bang C., Thum T. (2012). Novel techniques and targets in cardiovascular microRNA research. *Cardiovascular Research*.

[8] Kumar S., Kim C. W., Simmons R. D. (2014). Role of flow-sensitive microRNAs in endothelial dysfunction and atherosclerosis: mechanosensitive athero-miRs. *Arteriosclerosis, Thrombosis, and Vascular Biology*.

[9] Lee J., Lim S., Song B. W. (2014). MicroRNA-29b inhibits migration and proliferation of vascular smooth muscle cells in neointimal formation. *Journal of Cellular Biochemistry*.

[10] di Gregoli K., Jenkins N., Salter R. (2014). MicroRNA-24 regulates macrophage behavior and retards atherosclerosis. *Arteriosclerosis, Thrombosis, and Vascular Biology*.

[11] Zhang P., Zheng C., Ye H. (2014). MicroRNA-365 inhibits vascular smooth muscle cell proliferation through targeting cyclin D1. *International Journal of Medical Sciences*.

[12] Tsui N. B. Y., Ng E. K. O., Lo Y. M. D. (2002). Stability of endogenous and added RNA in blood specimens, serum, and plasma. *Clinical Chemistry*.

[13] Fichtlscherer S., Zeiher A. M., Dimmeler S. (2011). Circulating MicroRNAs: biomarkers or mediators of cardiovascular diseases?. *Arteriosclerosis, Thrombosis, and Vascular Biology*.

[14] Fichtlscherer S., de Rosa S., Fox H. (2010). Circulating microRNAs in patients with coronary artery disease. *Circulation Research*.

[15] Ren J., Zhang J., Xu N. (2013). Signature of circulating MicroRNAs as potential biomarkers in vulnerable coronary artery disease. *PLoS ONE*.

[16] Widera C., Gupta S. K., Lorenzen J. M. (2011). Diagnostic and prognostic impact of six circulating microRNAs in acute coronary syndrome. *Journal of Molecular and Cellular Cardiology*.

[17] Wang G.-K., Zhu J.-Q., Zhang J.-T. (2010). Circulating microRNA: a novel potential biomarker for early diagnosis of acute myocardial infarction in humans. *European Heart Journal*.

[18] Goren Y., Kushnir M., Zafrir B., Tabak S., Lewis B. S., Amir O. (2012). Serum levels of microRNAs in patients with heart failure. *European Journal of Heart Failure*.

[19] Corsten M. F., Dennert R., Jochems S. (2010). Circulating MicroRNA-208b and MicroRNA-499 reflect myocardial damage in cardiovascular disease. *Circulation: Cardiovascular Genetics*.

[20] Long G., Wang F., Duan Q. (2012). Human circulating microRNA-1 and microRNA-126 as potential novel indicators for acute myocardial infarction. *International Journal of Biological Sciences*.

[21] Tijsen A. J., Creemers E. E., Moerland P. D. (2010). MiR423-5p as a circulating biomarker for heart failure. *Circulation Research*.

[22] Ellis K. L., Cameron V. A., Troughton R. W., Frampton C. M., Ellmers L. J., Richards A. M. (2013). Circulating microRNAs as candidate markers to distinguish heart failure in breathless patients. *European Journal of Heart Failure*.

[23] Hsu A., Chen S. J., Chang Y. S. (2014). Systemic approach to identify serum microRNAs as potential biomarkers for acute myocardial infarction. *BioMed Research International*.

[24] Fihn S. D., Gardin J. M., Abrams J. (2012). ACCF/AHA/ACP/AATS/PCNA/SCAI/STS Guideline for the diagnosis and management of patients with stable ischemic heart disease: a report of the American College of Cardiology Foundation/ American Heart Association Task Force on Practice Guidelines, and the American College of Physicians, American Association for Thoracic Surgery, Preventive Cardiovascular Nurses Association, Society for Cardiovascular Angiography and Interventions, and Society of Thoracic Surgeons. *Journal of the American College of Cardiology*.

[25] Braunwald E., Antman E. M., Beasley J. W. (2002). ACC/AHA guideline update for the management of patients with unstable angina and non-ST-segment elevation myocardial infarction—2002: summary article: a report of the American College of Cardiology/American Heart Association Task Force on Practice Guidelines (committee on the management of patients with unstable angina). *Circulation*.

[26] Gidlöf O., Andersson P., Van Der Pals J., Götberg M., Erlinge D. (2011). Cardiospecific microRNA plasma levels correlate with troponin and cardiac function in patients with ST elevation myocardial infarction, are selectively dependent on renal elimination, and can be detected in urine samples. *Cardiology*.

[27] Li Y.-Q., Zhang M.-F., Wen H.-Y. (2013). Comparing the diagnostic values of circulating microRNAs and cardiac troponin T in patients with acute myocardial infarction. *Clinics*.

[28] Wang R., Li N., Zhang Y., Ran Y., Pu J. (2011). Circulating microRNAs are promising novel biomarkers of acute myocardial infarction. *Internal Medicine*.

[29] Long G., Wang F., Li H. (2013). Circulating miR-30a, miR-126 and let-7b as biomarker for ischemic stroke in humans. *BMC Neurology*.

[30] Li P., Zhang Q., Wu X. (2014). Circulating microRNAs serve as novel biological markers for intracranial aneurysms. *Journal of the American Heart Association*.

[31] Wei C., Henderson H., Spradley C. (2013). Circulating miRNAs as potential marker for pulmonary hypertension. *PLoS ONE*.

[32] Park M. Y., Herrmann S. M., Saad A. (2014). Circulating and renal vein levels of microRNAs in patients with renal artery stenosis. *Nephrology Dialysis Transplantation*.

[33] van Huyen J. P. D., Tible M., Gay A. (2014). MicroRNAs as non-invasive biomarkers of heart transplant rejection. *European Heart Journal*.

[34] Abue M., Yokoyama M., Shibuya R. (2014). Circulating miR-483-3p and miR-21 is highly expressed in plasma of pancreatic cancer. *International Journal of Oncology*.

[35] Hoekstra M., van der Lans C. A. C., Halvorsen B. (2010). The peripheral blood mononuclear cell microRNA signature of coronary artery disease. *Biochemical and Biophysical Research Communications*.

[36] Redell J. B., Moore A. N., Ward N. H., Hergenroeder G. W., Dash P. K. (2010). Human traumatic brain injury alters plasma microrna levels. *Journal of Neurotrauma*.

[37] Gu H., Li H., Zhang L. (2012). Diagnostic role of microRNA expression profile in the serum of pregnant women with fetuses with neural tube defects. *Journal of Neurochemistry*.

[38] van Rooij E., Sutherland L. B., Thatcher J. E. (2008). Dysregulation of microRNAs after myocardial infarction reveals a role of miR-29 in cardiac fibrosis. *Proceedings of the National Academy of Sciences of the United States of America*.

[39] Wu C., Gong Y., Sun A. (2013). The human MTHFR rs4846049 polymorphismincreases coronary heart disease risk through modifying miRNA binding. *Nutrition, Metabolism and Cardiovascular Diseases*.

[40] Olivieri F., Antonicelli R., Lorenzi M. (2013). Diagnostic potential of circulating miR-499-5p in elderly patients with acute non ST-elevation myocardial infarction. *International Journal of Cardiology*.

[41] Boon R. A., Iekushi K., Lechner S. (2013). MicroRNA-34a regulates cardiac ageing and function. *Nature*.

[42] Sayed A. S. M., Xia K., Yang T.-L., Peng J. (2013). Circulating microRNAs: a potential role in diagnosis and prognosis of acute myocardial infarction. *Disease Markers*.

[43] Udeanu M., Guizzardi G., di Pasquale G. (2014). Relationship between coronary artery disease and C-reactive protein levels in NSTEMI patients with renal dysfunction: a retrospective study. *BMC Nephrology*.

[44] Moreira D. M., da Silva R. L., Vieira J. L., Fattah T., Lueneberg M. E., Gottschall C. A. (2014). Role of vascular inflammation in coronary artery disease: potential of anti-inflammatory drugs in the Prevention of atherothrombosis: inflammation and anti-inflammatory drugs in coronary artery disease. *American Journal of Cardiovascular Drugs*.

[45] Wang H., Zhu H.-Q., Wang F., Zhou Q., Gui S.-Y., Wang Y. (2013). MicroRNA-1 prevents high-fat diet-induced endothelial permeability in apoE knock-out mice. *Molecular and Cellular Biochemistry*.

[46] Puri R., Nissen S. E., Shao M. (2014). Impact of baseline lipoprotein and C-reactive protein levels on coronary atheroma regression following high-intensity statin therapy. *American Journal of Cardiology*.

